# COVID-19 in South Korea

**DOI:** 10.1136/postgradmedj-2020-137738

**Published:** 2020-05-04

**Authors:** Jun Yong Choi

**Affiliations:** Department of Internal Medicine and AIDS Research Institute, Yonsei University College of Medicine, Seoul, South Korea

**Keywords:** infectious diseases

## Abstract

A novel coronavirus (severe acute respiratory syndrome-CoV-2) that initially originated from Wuhan, China, in December 2019 has already caused a pandemic. While this novel coronavirus disease (COVID-19) frequently induces mild diseases, it has also generated severe diseases among certain populations, including older-aged individuals with underlying diseases, such as cardiovascular disease and diabetes. As of 31 March 2020, a total of 9786 confirmed cases with COVID-19 have been reported in South Korea. South Korea has the highest diagnostic rate for COVID-19, which has been the major contributor in overcoming this outbreak. We are trying to reduce the reproduction number of COVID-19 to less than one and eventually succeed in controlling this outbreak using methods such as contact tracing, quarantine, testing, isolation, social distancing and school closure. This report aimed to describe the current situation of COVID-19 in South Korea and our response to this outbreak.

## Introduction

A novel coronavirus (severe acute respiratory syndrome (SARS)-CoV-2) that emerged from the city of Wuhan, China, in December 2019 has already caused a global pandemic and has been declared a public health emergency of international concern by the WHO.^1^ While this novel coronavirus disease (COVID-19) frequently induces mild symptoms, it has also generated severe illnesses among certain populations, including elderly individuals with underlying diseases such as cardiovascular disease and diabetes.^2^

As of 31 March 2020, a total of 9786 confirmed cases with COVID-19 have been reported in South Korea, including 162 deaths, 5408 recovered individuals released from isolation and 4216 patients staying in hospitals or non-hospital facility for isolation. In many countries such as the USA and European countries, the number of patients with COVID-19 is growing exponentially, and, at this point, South Korea is one of the few countries that have slowed the spread of COVID-19. This report aimed to describe the current situation of COVID-19 in South Korea and our response to this outbreak.

## Early stage of outbreak

The first imported case of COVID-19 was confirmed in South Korea on 20 January 2020.^3^ A 35-year-old woman who lived in Wuhan, China, arrived at the Incheon Airport on 19 January 2020. During the quarantine inspection process at the airport, her body temperature was reported as 38.3°C on a thermal scanner. She was hospitalised at a designated isolation hospital. Pan coronavirus conventional PCR assay was positive for the throat swab sample, and sequencing of the PCR amplicon showed that the sequence was identical to that of the 2019-nCoV isolated from the Wuhan patient.

Since the first case, cases imported from China and cases linked to the imported cases have been identified, and the sources of infection were traced by contact investigation until patient 29.^4^ Patient 29 was the first patient identified in Seoul who did not have an epidemiological link or travel history to China. From this patient, the possibility of community transmission was raised. As the number of confirmed cases was rapidly increasing, the Korean government raised the alert level from orange to red on 23 February 2020, resulting in the Ministry of Education ordering the closure of all schools and delaying the new school year opening by 1 week.

## Superspreading event and community transmission

**Figure 1:**
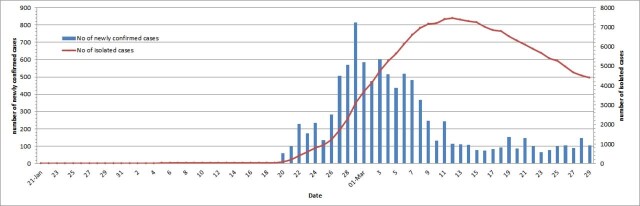
Trends of daily number of newly confirmed cases and isolated cases.

The epicentre of the COVID-19 outbreak in South Korea has been Daegu, a city of 2.5 million people, approximately 150 miles southeast of Seoul.^5^ The rapid spread of COVID-19 in South Korea is attributed to a superspreading event within a religious group called Shincheonji in the city of Daegu. This led to an explosive outbreak in the city of Daegu and Gyeongsangbuk-do. While the explosive outbreak has been controlled, sporadic spreading is still ongoing especially in mental health illness hospitals. The number of cases from Daegu and Gyeongsangbuk-do is attributed to 84% of all confirmed cases within South Korea as of 23 March 2020. Efforts in extensive testing, contact tracing, quarantine and isolation could effectively control the outbreak in these areas. However, a small number of outbreaks within some hospitals, including long-term care facilities, crowded facilities such as call centres, and household transmissions continues, and new cases imported from foreign countries such as European countries and the USA are emerging now. Figure 1 shows the daily trends of numbers of newly confirmed patients with COVID-19 and isolated patients with COVID-19 in South Korea. The number of newly confirmed cases was highest in 29 February 2020, and after that, the number gradually decreased until mid-March, but after 12 March, around 100 cases are steadily occurring every day. The number of patients in isolation was highest in 13 March 2020, but it has been decreasing ever since. This is because there are more patients who are cured and discharged than those who are newly isolated.

## Epidemiological characteristics

The Korea Centres for Disease Control and Prevention (KCDC) reported the basic epidemiological characteristics of 7755 patients with COVID-19 in South Korea as of 13 March 2020 using surveillance data retrieved from the KCDC-operated National Notifiable Disease Surveillance System.^6^ The female-to-male ratio was 62:38. The age group of 20–29 years accounted for 28.9% of all cases, followed by the age groups of 50–59 and 40–49 years. The case fatality proportion was 0.1% among the age groups of 30–39 and 40–49 years, then increased to 0.4% (50–59 years), 1.5% (60–69 years), 5.0% (70–79 years) and 8.5% (≥80 years). They described the characteristics of 66 fatal patients with COVID-19 as of 12 March 2020. The median age was 77 years (range 35–93 years), and the female-to-male ratio was 44:56. Of 63 cases, 96.8% were found to have coexisting conditions such as hypertension (47.6%), diabetes (36.5%), neurodegenerative disorders (16%) and pulmonary diseases (17.5%). The median interval between onset of symptom and death was 10 days (range 1–24 days), while the median interval between date of hospitalisation and the date of death was 5 days (range 0–16 days).

## Our responses

South Korea had experienced the Middle East respiratory syndrome coronavirus infection outbreak in 2015. At that time, nosocomial spread and superspreading events within hospitals mainly contributed to the outbreak.^7^ Since 2015, KCDC and many hospitals of South Korea have been prepared for the next outbreak of infectious diseases. The preparations done were with respect to healthcare personnel, facilities and the system as a whole. However, many experts now think that the preparations were not enough. Infectious diseases-specialised hospitals were not built, and the KCDC is still not independent from the Ministry of Health and Welfare. Although the number of negative pressure rooms in hospitals has been increased, the healthcare system in South Korea is still vulnerable to the outbreak of respiratory infections.

In the early stages of COVID-19 outbreak when imported cases from China and their linked cases were identified, KCDC actively performed contact tracing, quarantined the contacted persons, and diagnosed and isolated the COVID-19 cases as soon as possible. Some experts believe that entry from China should have been banned at an early stage of the outbreak, which the Korean government did not. After the SARS-CoV-2 emerged in China, KCDC rapidly developed tests according to the WHO guidelines and cooperated with diagnostic manufacturers to develop commercial test kits. The first test was approved on 7 February 2020, when the country had just a few cases, and it was then distributed to regional health centres. KCDC rapidly scaled up the diagnostic capacity within South Korea. Laboratory test for COVID-19 was initially performed at KCDC and then became available at 17 regional laboratories (Public Health and Environment Research Institute) throughout the nation, on 24 January 2020. Since 7 February 2020, the test facilities have been expanded to many laboratories, including tertiary hospitals, and more test centres were added later. Currently, 15 000–20 000 tests per day are being carried out by national central labs and 95 non-governmental clinical laboratories.^8^ These laboratories have been all certified by Korean Society for Laboratory Medicine and have also completed external quality assessments by the Korean Association of External Quality Assessment Service.

When the explosive outbreak in the city of Daegu and Gyeongsangbuk-do occurred in February, mass screening efforts identified patients with mild or no symptoms, and it contributed in controlling the cluster. For safe and efficient screening for COVID-19, drive-through screening centres have been designed and implemented in South Korea.^9^ The steps of the drive-through screening centres include registration, examination, specimen collection and instructions. Increased testing capacity of over 100 tests per day and prevention of cross-infection among testees in the waiting space seem to be the major advantages. A modelling study showed that South Korea has the highest diagnostic rate for COVID-19, and the diagnostic capacity of South Korea has been the major contributor in overcoming this outbreak. However, we cannot exactly estimate how many hidden cases are spreading COVID-19 at present.

The methods that can objectively verify the patient’s route claims, such as medical facility records, global positioning system, card transactions and closed-circuit television, were used for COVID-19 contact investigations in South Korea.^10^ The methods could provide accurate information on the location and time of exposure and details of the situation, thus reducing omissions in a patient’s route due to recall or confirmation bias that may have arisen from patient or proxy interviews.

During the early stages of COVID-19, all confirmed cases could be hospitalised into the designated isolation hospitals. However, when an explosive outbreak at the epicentre occurred, the lack of medical resources had become a reality. Healthcare professionals from other regions of South Korea went to the epicentre area to help out, while many critically ill patients were transferred to tertiary hospitals in other areas. The Korean government has opened several non-hospital facilities for the isolation of asymptomatic patients or patients with mild symptoms.

The basic reproduction number (R0) is an indicator for analysing and predicting the situation of an infectious disease, and the R0 of COVID-19 is around 2.5. This means that one patient spreads the disease to 2.5 during the period of transmission; if R0 is greater than 1, the infectious disease spreads gradually. We are trying to terminate the COVID-19 transmission chain and eventually succeed in controlling this outbreak with methods such as contact tracing, quarantine, testing, isolation, social distancing and school closure. A modelling analysis from the UK showed that these efforts for mitigation should last for months to control this outbreak temporarily; however, it remains to be seen whether these non-pharmaceutical interventions could be successful in controlling this outbreak in the long term.^11^ COVID-19 has unique characteristics. The R0 and mortality are higher than those of influenza, and the symptoms can be mild or absent even when the transmissibility is high. Many patients have developed pneumonia even though they have mild symptoms. These characteristics of the disease have shown us that it is a very difficult task to control this outbreak effectively. Maintaining the non-pharmaceutical interventions for a long time is a very challenging task for us.

It is very important to minimise mortality from this disease while reducing the R0 to less than 1. Because the mortality rate of COVID-19 is higher in elderly people and those with underlying diseases, they should be protected from this disease. In order to lower the mortality rate, inpatients who are vulnerable to COVID-19 should be protected as much as possible. Many hospitals in South Korea have quickly implemented systems to protect patients and medical staff, including outdoor triage clinics for patients with fever or respiratory symptoms; pre-emptive isolation wards for patients with pneumonia; entrance surveillance using fever checks and questionnaires about respiratory symptoms and travel history; programmes for protecting high-risk units such as the intensive care unit (ICU), haemodialysis unit, and operation rooms; and surveillance for hospital employees.

KCDC and other academic societies have issued a number of guidelines on diagnosis, treatment, infection control, quarantine and social distancing, and periodically update these guidelines. Korean scientists and medical doctors have been sharing their findings on COVID-19 globally through the latest and forthcoming publications.

Currently, there is no established standard antiviral treatment for COVID-19 other than supportive treatment. Based on the limited data, many experts in South Korea are trying to administer several antiviral agents at the judgement of the attending physicians. Most patients with COVID-19 have mild diseases, and those patients can usually be recovered without any antiviral treatments. The antiviral agents are used for patients with moderate or severe diseases with pneumonia or respiratory distress.

Table 1 shows the antiviral agents which are recommended for COVID-19 in South Korea and dosages of the antivirals for COVID-19.^12^ Lopinavir/ritonavir and hydroxychloroquine are the most commonly used antivirals for COVID-19 in South Korea. Remdesivir is available only for clinical trials.

**Table 1:** Dosages of antiviral agents for COVID-19

Medication	Normal renal function (CrCl >50 mL/min)	Impaired renal function (CrCl 25–50 mL/min)	Hemodialysis or CrCl <20 mL/min
Lopinavir/ritonavir	Lopinavir/ritonavir 400 mg/100 mg po every 12 hours	Same dose	Same dose
Hydroxychloroquine	Hydroxychloroquine 400 mg po 24 hours	Data not available	Data not available
Interferon-β1b	0.25 mg/mL subcutaneous injection qEOD	Data not available	Data not available
Remdesivir	200 mg loading dose on day 1 is given, followed by 100 mg intravenously once per day maintenance doses	Same dose	Same dose

CrCl, creatinine clearance; po, by mouth.

## Conclusion

KCDC; experts in infectious diseases, pulmonology, emergency medicine, laboratory medicine, infection control; healthcare providers; hospitals; and all Korean people are still trying hard to overcome the outbreak of COVID-19. The situation is changing rapidly as the outbreak evolves, so flexible evidence-based measures should be implemented for overcoming this outbreak. Although the rapid and sustained responses of South Korea to control the COVID-19 outbreak could slow the spread of the COVID-19 outbreak within this country, this outbreak could last for a long time, and at any time, unexpected surge may happen again.

It is too early to say whether our response has been successful. Our actions should be evaluated after the outbreak ends.

**Main messages**

- Various interventions such as contact-tracing, quarantine, testing, isolation, social distancing, and school closure are applied to control the COVID-19 outbreak in South Korea.
- South Korea has the highest diagnostic rate for COVID-19, which has been the major contributor in overcoming this outbreak.
- Although the rapid and sustained responses of South Korea to control the COVID-19 outbreak could slow the spread of the COVID-19 outbreak within this country, this outbreak could last for a long time, and at any time, unexpected surge may happen again.

**Current research questions**

- What is the most effective antiviral agent for COVID-19?
- How can you develop the effective vaccine for COVID-19?
- What is the most suitable strategies to control COVID-19 outbreak?

**Key references**

- Guan WJ, Ni ZY, Hu Y, *et al*. Clinical characteristics of coronavirus disease 2019 in China. *N Engl J Med* 2020 (doi: 10.1056/NEJMoa2002032).
- Kim JY, Choe PG, Oh Y, *et al*. The first case of 2019 novel coronavirus pneumonia imported into Korea from Wuhan, China: implication for infection prevention and control measures. *J Korean Med Sci* 2020;35(5):e61.
- Korean Society of Infectious Diseases. Report on the epidemiological features of coronavirus disease 2019 (COVID-19) outbreak in the Republic of Korea from January 19 to March 2, 2020. *J Korean Med Sci* 2020;35(10):e112.
- Kim YJ, Sung H, Ki CS, et al. Covid-19 testing in South Korea: current status and the need for faster diagnostics. *Ann Lab Med* 2020;40:349-50
- Kwon KT, Ko JH, Shin H, *et al*. Drive-through screening center for COVID-19: a safe and efficient screening system against massive community outbreak. *J Korean Med Sci* 2020;35(11):e123.

**Self-assessment questions**

- While this novel coronavirus disease (COVID-19) frequently induces mild symptoms, it has also generated severe illnesses among certain populations including elderly individuals with underlying diseases such as cardiovascular disease and diabetes.
- South Korea has the highest diagnostic rate for COVID-19, which has been the major contributor in overcoming this outbreak.
- The basic reproduction number (R0) is an indicator for analysing and predicting the situation of an infectious disease, and the R0 of COVID-19 is around 2.5.
- Patients with COVID-19 with severe pneumonia have higher transmissibility than patients with mild diseases.
- Currently, there is no established standard antiviral treatment for COVID-19 other than supportive treatment.

**Answers**

- True.
- True.
- True.
- False.
- True.
